# A Gateway Framework to Guide Major Health System Changes: Comment on "‘Attending to History’ in Major System Change in Healthcare in England: Specialist Cancer Surgery Service Reconfiguration"

**DOI:** 10.34172/ijhpm.2023.7681

**Published:** 2023-03-12

**Authors:** Kathy Eljiz, David Greenfield, Alison Derrett

**Affiliations:** ^1^University of New South Wales, Sydney, NSW, Australia; ^2^Western Sydney Local Health District, Sydney, NSW, Australia

**Keywords:** Major System Change, Restructuring, Health Systems, Healthcare Safety, Healthcare Quality

## Abstract

Recent events – on both a global scale and within individual countries – including the lockdowns associated with COVID-19 pandemic, inflation concerns, and political tensions, have increased pressure to reconfigure social services for ongoing sustainability. Healthcare services across the world are undergoing major system change (MSC). Given the complexity and different contextual drivers across healthcare systems, there is a need to use a variety of perspectives to improve our understanding of the processes for MSC. To expand the knowledge base and develop strategies for MSC requires analysing change projects from different perspectives to distil the elements that drove the success. We offer the Gateway Framework as a collaborative transformational system tool to assess and reorganise operations, services, and systems of healthcare organisations. This framework and guiding questions, accounts for past events whilst being proactive, future orientated, and derived from externally defined and a standardised requirements to promote safe, high-quality care.

## Introduction

 Healthcare policy-makers, leaders, managers, clinicians, and consumers focus over time has expanded to include outcome measures, alongside inputs and processes, to improve care delivery and experiences.^1^ Recent events — on both a global scale and within individual countries — including the lockdowns associated with COVID-19 pandemic, inflation concerns and political tensions, have further increased pressure to reconfigure social services for ongoing sustainability.^2^ Consequently, in many countries, healthcare services are undergoing major system change (MSC).^3,4^ Hence, Perry and colleagues^5^ work seeking to understand how to effectively achieve MSC — enablers, barriers, processes and outcomes — is relevant and vital for healthcare organisations everywhere.

 Perry and colleagues draw attention to the need to use a variety of perspectives to improve our understanding of the processes for MSC. They argue that when designing and implementing change processes, leaders should be “aware of both documented history and accounts of history from key stakeholders, as well as considering their power within the system” (p. 12). They clearly outline how an examination of stakeholder perspectives of major governance and leadership decisions involved in previous change attempts identifies messages that can be categorised into three key lessons: how past experience can, and should be, used to facilitate the reconfiguration of health services; how clear governance arrangements and leadership structure can encourage engagement with diverse stakeholders; and, the need for clear outcomes measures to be fed back to communicate change progress/success.^5^ These recommendations are reflective of the current knowledge base of the broader change management literature.^6-9^

 To expand the knowledge base and develop strategies for MSC requires analysing change projects from different perspectives to distil the elements that drove the success.^4^ There is an opportunity and need to merge change management with safety and quality, improvement and implementation methodologies.^9^ We compliment the work of Perry and colleagues, and expand the approaches for understanding MSC, by offering a distinct perspective of investigating MSC through a combination of safety and quality and transformational system thinking lenses, which we label the ‘Gateway Framework.’ This framework, accounts for past events whilst being proactive, future orientated, and derived from externally defined and standardised requirements to promote safe, high-quality care. Furthermore, this approach aligns professionals’ internal motivations for improvement and collaboration to move past historical practices^4^; the framework is a ‘gateway’ to an improved future. In doing this, the study seeks to answer the following research question: how can we more effectively assess and reorganise operations, services, and systems of healthcare organisations, aligning activities with safety and quality requirements? We address this question we apply the Gateway Framework to the case study of Perry and colleagues to demonstrate applicability and the different insights generated.

## The Gateway Framework: A Tool for Generating Heath System Transformation

 The Gateway Framework is a transformational system tool with five elements that encompassing governance systems, stakeholder engagement, professional collaboration and practices, and enacting patient-centred care (Table, Figure). A first step is: defining the focus of attention — is it a service, department, division or organisation?; and identifying key stakeholders — professional, administrative, community and policy-makers? The Gateway Framework is then used with the stakeholders as a set of guiding questions to direct the analysis and development of change management plans for improvement. The applied questions are challenging and designed to illicit what has occurred, what now is happening and what needs to be implemented. The guiding questions have been derived from the National Model Clinical Governance Framework (NMCGF)^10^ and empirical knowledge about system level changes, such as large scale health facility redevelopment.^11^ By linking the Gateway Framework to a national level safety and quality framework, there is an alignment to the systems approach and reflects the importance of governance, leadership and outcome measures outlined in the article by Perry and colleagues. The NMCGF builds on the National Safety and Quality Health Service Standards,^10^ and reflects similar elements used in other national programs and frameworks regulating safety and quality, this includes: the Institute of Medicine Framework and the Institute for Health Improvement Framework for Safe, Reliable, and Effective Care (USA); the Canadian Quality and Patient Safety Framework Evaluation (Canada); and the Scottish National Performance Framework (UK).

**Table:** The Gateway Framework Elements and Guiding Questions

**Element **	**Guiding Questions **
Governance systems	What has been the governance arrangements? What has been their strengths and limitations? Have there been major failures or problems? What legal/regulation/policy directives need to be complied with? What major governance changes have occurred in the past X* years? (*X to be defined by change agent accounting for team/service/organisational focus) What are the internal and external drivers for the changes? What are the cultural factors dominant at organisational, service and team levels? What financial and clinical resources are required? What boards, committees and groups are needed to drive the changes?
Stakeholder engagement	How is stakeholder engagement conducted by the organisation? Is it effective? How is it evaluated/measured/assessed? Who are our internal and external stakeholders and which of these need to be engaged? How will they be engaged? What avenues are required to engage a multitude of stakeholders? What internal and external feedback mechanisms will be deployed?
Performance and collaboration	Are there existing systems that promote performance and collaboration? How is “performance” understood and measured? Does it have an individual, team and/or department focus to it? What value is placed on individual, team, and organisational outcomes? What is more/less valued? How is “collaboration” understood and measured? Is collaboration an intraprofessional and/or interprofessional activity? What needs to change for clinical, administrative, and corporate professionals to work more effectively together to overcome traditional silos? What needs to change for clinical, administrative, and corporate professionals to work more effectively together to promote a safety culture?
Physical environment/Built environment	Does the physical space enable or hinder the delivery of care? What are the enablers and barriers? What physical space changes would positively reinforce collaborative care delivery models? How can patient management and flow be improved? How can the physical space – quieter, private, calming – promote safer care interactions?
Patient-centred care	How is “patient-centred care” understood and measured? How does the “patient” fit into patient-centred care? Are they involved in planning and decision making? What changes need to be made for more effective engagement and participation of patients and the community? How would they be measured and evaluated? Is patient-centred care consistent with how performance and collaboration are understood and measured? If not, what are the differences? How can they be brought into alignment? What new models of patient-centred care are needed for more effective collaboration? What does success look like, and how will success be measured?

**Figure:**
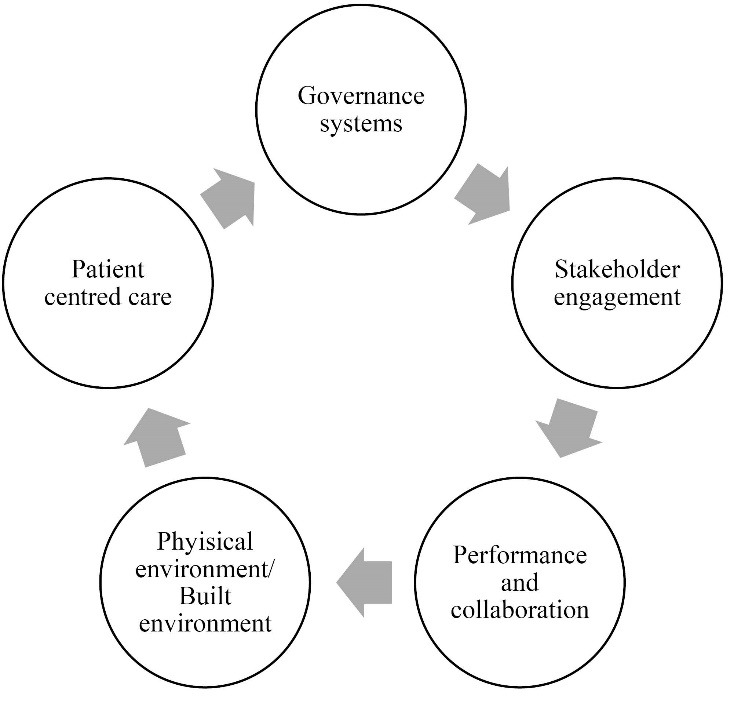
The Gateway Framework.

 The Gateway Framework is a tool to be utilised in an iterative manner, with analysis moving back and forth across the five elements using the guiding questions as starting points to direct thinking, review and investigation. The tool is to be used to generate collaboration within and across the stakeholders. The aim is to generate a system change plan that identifies enablers, barriers, processes and outcomes. In generating the questions, we firstly drew on the expertise of the study team in accreditation, operational and strategic expertise in running healthcare organisations, and quality and safety, regulation and organisational development research. We did this by applying an iterative process of reviewing the NMCGF and associated resources^12^ and the academic literature. Second, the questions derived were discussed with academic colleagues and healthcare industry executives to ensure credibility and validity.

## MSC Transformation: Analysis to Determine a Direction for Change

 We provide additional insight into how the Gateway Framework questions can be used to assist with healthcare MSC. We do so by reviewing the key findings or lessons from Perry and colleagues through the Gateway Framework; ideas from Perry and colleagues are linked to the NMCGF and together are used to generate questions for use in safety and quality improvement efforts.

## Governance Systems

 Governance, leadership and culture are important drivers of MSC; having an in-depth understanding of the historical systems, current systems and details associated with these elements are essential to planning for change. A review of documentation, personal accounts, and external national benchmarking reports can help obtain accurate information on accountability arrangements, prior change initiatives and outcomes. Knowledge of the local context, including previous attempts at MSC, and associated outcomes can assist with planning future MSC. Outcomes can include clinical and procedural or process, with external feedback loops to communicate changes to diverse stakeholder groups. The major resources allocated, such as financial and clinical, can provide a good indication of investment versus outcome, and help shape future efforts. Formal and informal oversight is necessary and can be provided through boards, trusts, committees and local groups representing management, operational staff and the broader community. Reviewing reporting requirements to state and regulatory agencies will assist to ensure external alignment with governance and care standards or expectations are maintained.

## Stakeholder Engagement

 Ownership of change is crucial for ongoing implementation of new ways of working; this outcome can be achieved by meaningful engagement with all parties affected by the change. In particular, early engagement in the planning phase with all stakeholders – frontline clinicians, managers, administrative staff and consumers – can help overcome historical barriers to change. Stakeholder engagement is effective when integrating designated personnel – those that lead reforms - with distributed personnel – those that enact reforms. The addition of clinicians external to the organisation and patient advocates – stakeholders impacted by the organisation – thus providing a balanced, representative view into the future direction. Additionally, to embed the change in the longer term, ensure consistent information sharing and reciprocal advice across internal and external stakeholders throughout all stages of change. What is meaningful and relevant information will vary from setting to setting, hence both designated and distributed personnel require an understanding of contextual factors (local drivers) in different organisational spaces. This can be attained via feedback before, during and after the MSC through internal and external mechanisms, including via regulatory and clinical standards.

## Performance and Collaboration

 Challenging traditional competition through promoting cooperation across professions, functions and departments can assist with redefining and enhancing performance at all levels. Stakeholder engagement, involving the end users which includes patients and distributed personnel (staff), in the development is a concrete strategy to encourage inter- and intra- professional collaboration for improved outcomes. Ensuring there are tangible outcome measures will assist all involved understand how performance is monitored and measured. By promoting distributed ownership of services, such cancer services, a positive feedback loop can be established; shared responsibility by teams redefines how models of care are conceptualised, operationalised and subsequentially managed. Individual and team behaviours and performance outcomes become aligned and interdependent.

## Physical Environment/Built Environment

 Distributed personnel need opportunity to review how the physical space and other contextual factors promotes or hinders service specifications, models of care, cooperation behaviours and outcomes. Identifying enablers and barriers to required behaviours in the current space is for the purpose of using this knowledge for defining the new environment. The future goal being to have the space direct the required positive model of care with integrated cooperative behaviours and outcomes. Designated personnel need to guide discussion with distributed personnel about how technological, environmental and ideological factors will change and enable the physical context to be quieter, more private, and calming for patients and staff. Thus, enabling safer, high quality care outcomes.

## Patient-Centred Care

 Patient-centred care is achieved when aligned to organisational performance and collaboration requirements and outcome measures that address patient/consumer issues. Designated and distributed personnel both need to understand and minimise negative power dynamics when planning and delivering services. Key to a successful transformation process is ensuring patient and family involvement in planning and decision making. To enable the community to become more engaged requires developing their health literacy. A key question to engage all stakeholders in answering is: what does success look like, and how will success be measured?

## Conclusion

 MSC is contingent upon examining the elements that led to positive outcomes during change projects. Plans and strategies to achieve MSC can be informative, wide-ranging and comprehensive when investigated from diverse perspectives. In addition to the governance and accountability focus adopted by Perry and colleagues, the Gateway Framework – a combined safety and quality, improvement transformative systems thinking lens - offers a complementary investigative view. Through an applied set of guiding questions, the lens accounts for past events whilst being future oriented and promoting safe, high-quality care. Used within a collaborative diverse stakeholder context, the lens can be applied to assess and reorganise operations, services, and systems of healthcare organisations. Further research is required to investigate the best ways of using the Gateway Framework and guiding questions. The aim is to generate a transformative system change plan, aligned to safety and quality requirements, that identifies enablers, barriers, processes and outcomes.

## Ethical issues

 Not applicable.

## Competing interests

 Authors declare that they have no competing interests.

## Authors’ contributions

 KE and DG designed and scoped the manuscript. All authors undertook research, writing and editing of the manuscript.
